# Monitoring Processes in Visual Search Enhanced by Professional Experience: The Case of Orange Quality-Control Workers

**DOI:** 10.3389/fpsyg.2018.00145

**Published:** 2018-02-14

**Authors:** Antonino Visalli, Antonino Vallesi

**Affiliations:** ^1^Department of Neuroscience, University of Padova, Padua, Italy; ^2^San Camillo Hospital IRCCS, Venice, Italy

**Keywords:** real-world cognitive enhancement, visual search, expertise, cognitive control, professional training

## Abstract

Visual search tasks have often been used to investigate how cognitive processes change with expertise. Several studies have shown visual experts' advantages in detecting objects related to their expertise. Here, we tried to extend these findings by investigating whether professional search experience could boost top-down monitoring processes involved in visual search, independently of advantages specific to objects of expertise. To this aim, we recruited a group of quality-control workers employed in citrus farms. Given the specific features of this type of job, we expected that the extensive employment of monitoring mechanisms during orange selection could enhance these mechanisms even in search situations in which orange-related expertise is not suitable. To test this hypothesis, we compared performance of our experimental group and of a well-matched control group on a computerized visual search task. In one block the target was an orange (expertise target) while in the other block the target was a Smurfette doll (neutral target). The a priori hypothesis was to find an advantage for quality-controllers in those situations in which monitoring was especially involved, that is, when deciding the presence/absence of the target required a more extensive inspection of the search array. Results were consistent with our hypothesis. Quality-controllers were faster in those conditions that extensively required monitoring processes, specifically, the Smurfette-present and both target-absent conditions. No differences emerged in the orange-present condition, which resulted to mainly rely on bottom-up processes. These results suggest that top-down processes in visual search can be enhanced through immersive real-life experience beyond visual expertise advantages.

## Introduction

Many daily activities require us to search around in order to locate particular items, such as an icon on a messy computer desktop or a friend in a crowded bar. Search performance depends on several factors, some involving the perceptual properties of stimuli and search contexts (Treisman and Gelade, 1980; Duncan and Humphreys, 1989), and others the observer and her/his previous experience (e.g., past knowledge about or affective attachment to the stimuli, Biggs et al., 2012). As far as the observer's factors are regarded, previous studies have investigated how expertise affects object recognition and detection. Several studies report that experts in particular topics (e.g., birds, cars or fingerprints) are faster in discriminating objects pertaining to their area of expertise (Gauthier et al., 2000; Busey and Vanderkolk, 2005; Curby and Gauthier, 2009; Bukach et al., 2010). Moreover, other studies suggest that this facilitation can potentially extend to other visual processes, such as categorization (e.g., recognizing images of expertise from fragments; Harel et al., 2011) and detection (e.g., localizing targets of expertise among distractors from non-expertise categories or in natural scenes; Golan et al., 2014; Reeder et al., 2016).

An interesting case of expertise involves professional searchers. Radiologists, proofreaders, or airport security screeners take advantage not only of their domain-specific knowledge (e.g., knowledge of tumors, spelling errors, weapons, respectively), but also of their task-specific training. In a study with Transportation Security Administration Officers, Biggs et al. (2013) investigated whether professional searchers' expertise could influence visual search performance beyond the task they have been trained on. Comparing professional and non-professional searchers, the authors found differences in visual search strategies. More in detail, while search speed explained most accuracy variance in non-professional and early-career professional searchers, search consistency (i.e., trial-to-trial RT variability) was the best accuracy predictor in experienced professional searchers. The authors concluded that the effects of professional training and experience were likely extended to generalized search behaviors. Interestingly, since consistent search behaviors may allow a more efficient use of cognitive resources, the authors highlighted the importance of top-down control in visual search performance. In this regard, Harel (2016) describes visual expertise as an interactive process that emerges from enhanced interactions between the visual system and multiple top-down mechanisms including attentional control, domain-specific knowledge, and task-specific strategies.

Summarizing, previous research seems to suggest that visual expertise most likely reflects an enhanced engagement of multiple interactive processes that experts manifest with their objects of specialization (Harel, 2016). However, in this study we wanted to go beyond domain-specific aspects by addressing the following question: does intense visual search experience enhance top-down control mechanisms independently of the nature of the target? Indeed, professional experience may influence search behaviors in several ways independently of visual expertise. As mentioned above, professional experience can improve search behaviors through the acquisition of more efficient search strategies, such as search consistency (Biggs et al., 2013; Biggs and Mitroff, 2014). Here, we further explored a possible influence of expertise by testing the hypothesis that intense professional search experience could boost top-down control processes involved in generalized visual search behaviors.

Specifically, we focused on monitoring mechanisms, a series of “quality check” processes that aim to optimize behavior (cf. Stuss and Alexander, 2007; see Vallesi, 2012 for an overview). Monitoring skills are required in many cognitive domains and task contexts. For example, it has been shown that participants monitor the elapse of time during a variable foreperiod task (Vallesi et al., 2014; Capizzi et al., 2015), their performance to successfully detect errors (Ullsperger et al., 2014), the occurrence of critical events (Capizzi et al., 2016; Tarantino et al., 2017), or their progress toward a desired goal (Benn et al., 2014). Most germane to our study, monitoring is also involved in visual search paradigms that require checking and evaluating the presence/absence of a target embedded among distractors. When the target is absent, monitoring should intervene more strongly than when the target is present and clearly detectable (Vallesi, 2014). This is because while the detection of a present salient target is mainly driven by bottom-up processes that automatically attract participants' attention (Treisman and Gelade, 1980), determining the absence of a target needs a more extensive and wider inspection of the search array.

In order to test the role of expertise in the monitoring mechanisms during visual search, we compared a group of quality-control employees working on the orange production line of some citrus farms and a well-matched control group. The daily job of these quality-control workers consists of many hours spent inspecting and evaluating oranges rolling down a conveyor belt, and discarding the oranges perceived as not suitable on the basis of visual features such as size, color, or skin imperfections that worsen their organoleptic properties. Quality-controllers were selected as the experimental group since they routinely perform a job that extensively engages monitoring processes, which as a result should improve such processes in visual search independently of the nature of the target. All participants performed two blocks of a visual search task with images of several objects. In one block the target was an orange while in the other block was a Smurfette doll. If expertise advantages emerged only with objects of expertise, we would expect to find quality-controllers to be faster than control participants just with the orange target. On the contrary, we predicted better controllers' performance not only with the orange target, but in all the situations in which monitoring is especially involved (Weidner et al., 2009), mainly when the target is absent (Vallesi, 2014).

## Materials and methods

### Participants

Twenty-four quality-control employees on the production line of orange fruits (12 women; mean age: 51.2 years, *SD* = 9.4, range: 25.5–65.9 years; mean education: 8.5 years, *SD* = 2; hereafter referred to as quality-controllers) and 23 control participants (13 women; mean age: 51.3 years, *SD* = 9.9, range: 24.7–64.7 years; mean education: 8.6 years, *SD* = 1.7), all recruited in Sicily, Italy, voluntarily took part in the study. Quality-controllers reported to work about 6–8 h in a day, for about 6–9 months per year. Mean working experience was 13.9 years (*SD* = 8.2, range: 3–35). All participants reported to have normal or corrected-to-normal visual acuity and normal color vision. The two groups were equivalent in age [*t*_(45)_ = 0.057, *p* = 0.955], education [*t*_(45)_ = 0.199, *p* = 0.843], and sex [Yates' corrected χ^2^(1, *N* = 47) = 0.024, *p* = 0.876]. One further control participant was excluded from analysis due to poor task performance. The procedures involved in this study were approved by the Comitato Etico per la Sperimentazione dell'Azienda Ospedaliera di Padova. Participants gave their written informed consent, in accordance with the Declaration of Helsinki, and they were reimbursed 25 euros for their time.

### Stimuli and design

**Figure 1:**
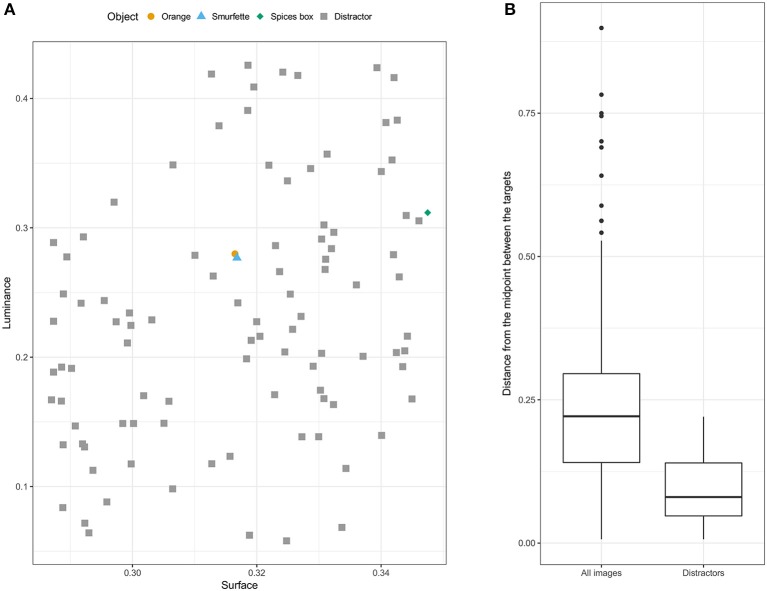
Luminance and surface characteristics of targets and distractors. **(A)** Representation of the positions of selected targets and distractors in the [luminance, surface] space. **(B)** The two boxplots show the Euclidian-distance distributions of all ALOI images (left) and of the selected distractors (right) from the midpoint between the two targets in the [luminance, surface] space.

The visual search task was implemented in Matlab using the Psychophysics Toolbox (Brainard and Vision, 1997; Kleiner et al., 2007) and presented on a Dell Intel Core laptop computer. Participants sat facing the screen at a viewing distance of approximately 60 cm. Stimuli were 100 objects selected from the 1,000 images of the ALOI (Amsterdam Library of Object Images; Geusebroek et al., 2005) database. The image of an orange was selected as target for one of the two experimental blocks. The other target was selected based on matched luminance and surface area characteristics. Namely, for each ALOI image the mean luminance across all pixels (defined as L dimension in the CIE L^*^a^*^b^*^ color space) and the object surface area (i.e., amount of pixels) were computed. A bi-dimensional Euclidian space was constructed, with Min-Max scaled luminance and surface (i.e., both measures brought into the range [0 1]) as dimensions. The image with the smallest distance from the orange in the luminance-surface Euclidian space was selected as the second target, that is, the Smurfette doll (Figure 1A). Ninety-seven images with low luminance-surface Euclidean distance from the targets were pseudorandomly selected as distractors. Specifically, they were chosen if located at a distance <0.221 (median distance of ALOI images from the midpoint between the two targets). The final set of 97 distractors (Figure 1B) had a median distance of 0.080 (IQR = 0.220). One additional image (a spicy box) with low distance from the targets (distance = 0.04) was pseudorandomly selected as target for the practice block.

The task had a 2 × 3 × 2 factorial design with target type, array size, and target presence as factors. The target type (orange vs. Smurfette) was manipulated between blocks and the order of blocks counterbalanced across participants. The search array consisted of 12, 24, or 48 object images (2.66° × 2.00° of visual angle) with transparent background displayed against a middle gray background. The array was arranged in a grid of 6 × 8 available locations, each of which subtending a visual angle of 3.66° × 2.25°. To perturb this grid-like arrangement and prevent a line-by-line search, on each trial object locations were randomly jittered by a maximum of 0.25° horizontally and 0.5° vertically (Hershler and Hochstein, 2009).

**Figure 2:**
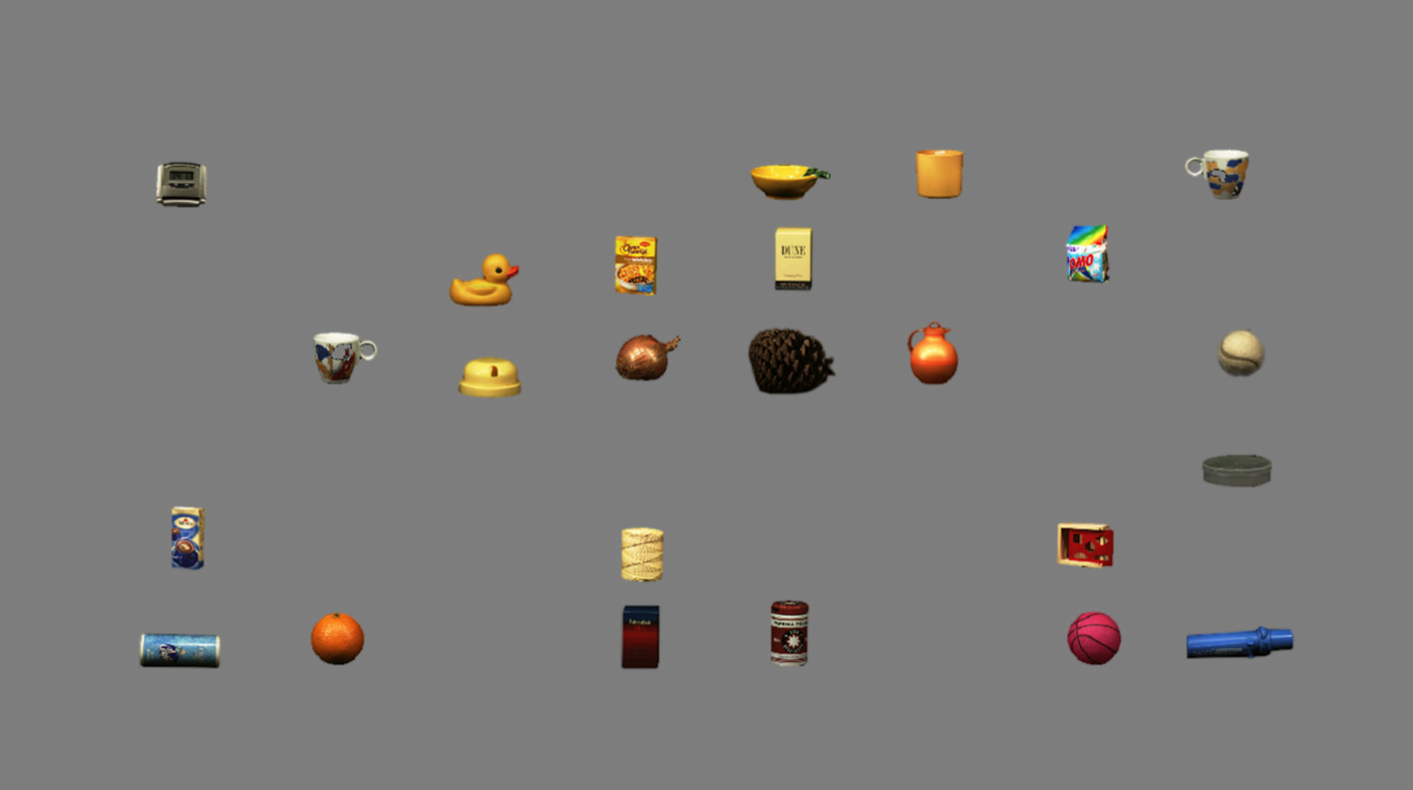
Example of a trial display (orange-present condition; array size: 24).

Each experimental block consisted of 288 trials. At the beginning of each block, the target was presented for as long as participants demanded to memorize it. Next, on each trial a fixation cross was displayed at the center of the screen for an interval randomly jittered between 0.75 and 1.5 s to make the onset of stimuli (equally) unpredictable. The cross was then replaced by an array of object images (Figure 2) displayed until participant's response. Within blocks, each combination of array size × target presence was presented 48 times in pseudorandom order. For every array size, each of the 48 available positions was occupied in turn by the target, while distractors were pseudo-randomly assigned to one of the remaining locations. Participants were required to press one of two response keys (“Z” or “M”) to indicate whether the target was present or not. The assignment of the two response keys to either target presence or absence was counterbalanced across participants. Participants were instructed to be as fast as possible, but also accurate. A low tone was provided after errors. A practice block of 12 trials preceded the two experimental blocks. After each practice trial, feedback on accuracy and speed was provided: either a green tick for correct responses or a red cross for wrong responses given in less than 5 s, or “Try to be faster …” for response times (RTs) longer than 5 seconds (threshold based on previous findings from similar visual search tasks: Hershler and Hochstein, 2005, 2009; VanRullen, 2006; Golan et al., 2014).

### Statistical analyses

All statistical analyses were performed using R (R Core Team, 2016). Trial-level measures (i.e., single-trial log-transformed RT and dichotomous accuracy) were analyzed by conducting mixed-effects models using the lme4 library (Bates et al., 2014). Mixed-effects modeling has several advantages over traditional general linear model analyses (such as repeated-measures ANOVA) that make it suitable for trial-level measures (Baayen et al., 2008; Quené and van den Bergh, 2008). First, since mixed-effects model analyses are conducted on trial-level data (i.e., they do not require prior averaging of participant's data to a single value per experimental condition), they allow preserving and taking into account any variability across individuals, thus increasing the accuracy and generalizability of the parameter estimate. Moreover, for the same reasons, they account for intrinsic unreliability of participant's average scores due to differences in intra-individual performance variability (Kliegl et al., 2011). Another advantage of mixed-effects modeling over repeated-measures ANOVA is that it is not restricted to predictors with categorical levels, but it easily allows to test for the effect of discrete/continuous variables and their interactions with categorical variables, usually with a gain in statistical power (Kliegl et al., 2011). Especially concerning RTs, a further advantage is the possibility to control for many longitudinal effects during the task. First, there are the effects of learning and fatigue (Baayen et al., 2008). Second, the response in a trial is usually heavily influenced by what happens in the preceding trial (for example RT in the preceding trial is often a good predictor of RTs, Baayen et al., 2008). Using mixed-effects models, all these sources of experimental noise are easily brought under statistical control. Additionally, since mixed-effects models have been extended to generalized linear models, they can be used to efficiently model dichotomous data, such as accuracy in our task (Quené and van den Bergh, 2008).

Summary variables, such as Signal Detection Theory measures or item image properties, were analyzed using standard general linear model analyses (such as repeated-measures ANOVA or *t*-test).

## Results

### Accuracy

Response accuracy at each trial, given its dichotomic nature, was analyzed by conducting a Generalized Linear Mixed Model (GLMM) with logit link function using the *glmer* function from the lme4 library (Bates et al., 2014). Log-transformed RTs, target presence, target type, array size, and group (with their interaction terms) were entered into the model as fixed effects. A random intercept varying among participants and among response bias (C) within participants, as well as uncorrelated random intercept and slope for trial order were entered into the model as random effects (an R-notation formula of the model is presented in Equation 1). In order to facilitate the convergence of the models, continuous variables (i.e., array size, log RT, and trial order) were scaled and centered within each participant using the R function scale.

$$\begin{array}{l} accuracy\;\sim \;trial\;+\;\log RT\;+\;presence\;*\;target\;*\;array\;*\;group\\ +\left(1|id/c\right)+\left(0+trial|id\right) \end{array}$$

Response bias, from Signal Detection Theory, was computed for each combination of target presence, target type, and array size, and defined as *C* = −0.5^*^(Z_Hit_+Z_FA_), where Z_Hit_ and Z_FA_ are the standardized hit rates and false alarm rates, corrected as indicated by Snodgrass and Corwin (1988). C was introduced since it influences the probability of responding present/absent in visual search tasks (Palmer et al., 2000). Uncorrelated random intercept and slope for trial order were introduced to control for possible effects of learning and fatigue. The log- transformed RT for each trial was included to control for possible speed-accuracy trade-off effects. To explore the influence of group on accuracy, we first compared the model without fixed effects (i.e., null model; Macc0) with the model containing the predictor group. The likelihood ratio test showed that group did not significantly improve the model fit [$\chi_{\left(1\right)}^{2}$ = 0.01, *p* = 0.971], suggesting that accuracy did not change across groups. We explored the influence of the other predictors by incrementally adding each of them (with their interaction terms) to the null model. Table 1 shows the results of the likelihood ratio test. The model Macc5, which included all the fixed effects (and their interaction terms) with the exception of group, resulted the best model to explain accuracy data distribution. In contrast, the inclusion of group and its interaction terms did not significantly increase the goodness-of-fit of the model. No other variable (e.g., pre-accuracy) significantly improved the fitting of the model. Marginal R^2^ (Johnson, 2014) of Macc5, which represents the variance explained by the fixed effects, was 0.15; conditional R^2^, which is the variance explained by both fixed and random effects, was 0.21.

**Table 1:** Model fit analysis for accuracy data.

	**Fixed effects**	**Model *df***	**Chisq (*df*)**	***p***	**AIC**	**ΔAIC**	**RL**
M_acc_0	Intercept	4			7,447		
M_acc_1	Trial	5	17.19 (1)	<0.001	7,432	15.19	>10^3^
M_acc_2	Trial + logRT	6	45.62 (1)	<0.001	7,388	43.62	>10^9^
M_acc_3	Trial + logRT + presence	7	335.13 (1)	<0.001	7,055	333.13	>10^72^
M_acc_4	Trial + logRT + presence × target	9	183.84 (2)	<0.001	6,876	179.84	>10^39^
M_acc_5	Trial + logRT + presence × target × array	13	71.97 (4)	<0.001	6,812	63.97	>10^13^
M_acc_6	Trial + logRT + presence × target × array × group	21	6.36 (8)	= 0.606	6,821	−9.64	0.008

*Fixed effects and degrees of freedom (df) are reported for each model. Models were fitted using maximum likelihood. Chi-squared and degrees of freedom, Chisq (df), and probability value, p, are based on the likelihood ratio test between successive models. ΔAIC indicates the difference in Akaike Information Criterion (Burnham and Anderson, 2002) between the model M_acc_(n−1) and the model M_acc_(n) [e.g., for the model M_acc_3, ΔAIC = AIC(M_acc_2) – AIC(M_acc_3)]. To compare the relative evidence of each model, we computed the relative likelihood, RL = exp(ΔAIC/2)*.

**Figure 3:**
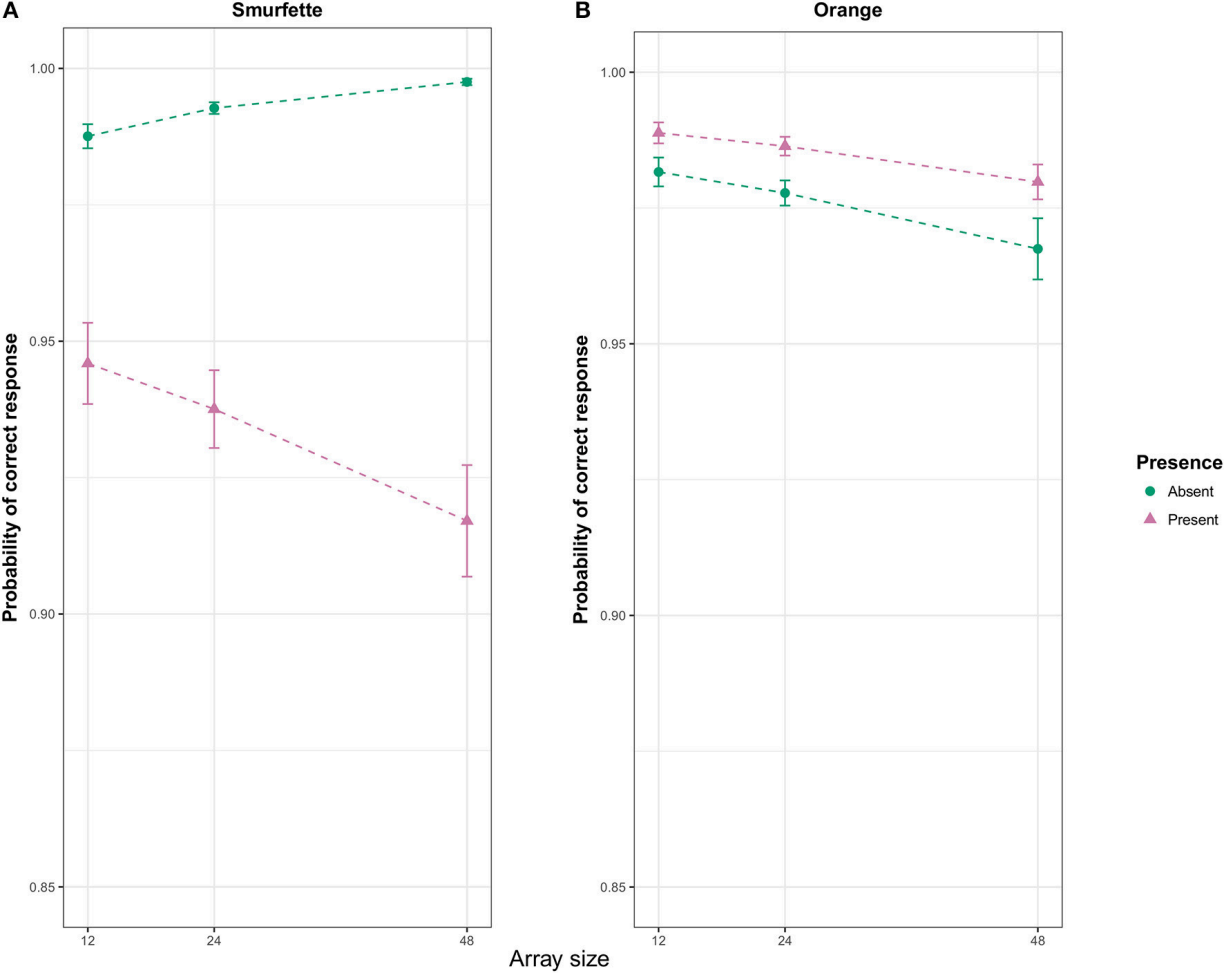
Effect display (Fox, 2003) for the interaction of presence and array size in the Smurfette **(A)** and orange **(B)** GLMMs fit to response accuracy data. Error bars represent standard errors of the estimated effect.

The Wald test (Wald, 1945) on the final model, Macc5, revealed a number of significant effects. For these effects, we report the estimated coefficient (b), the associated standard error (SE) and the z-statistics (z). A significant interaction was found between the predictors target presence, target type, and array size (*b* = −0.59, *SE* = 0.17, *z* = −3.45, *p* < 0.001). To interpret this three-way interaction, two GLMMs were fitted on the two task blocks separately. In the Smurfette block, the Wald test revealed a significant main effect of target presence (*b* = −2.44, *SE* = 0.13, *z* = −18.86, *p* < 0.001), with lower accuracy in the Smurfette-present than absent condition (Figure 3A). This effect was modulated by the array size (interaction: *b* = −0.87, *SE* = 0.12, *z* = −6.95, *p* < 0.001). In particular, the difference between present and absent conditions increased with increasing array size (Figure 3A). In the orange block, the Wald test revealed a significant main effect of target presence (*b* = 0.50, *SE* = 0.14, *z* = 3.62, *p* < 0.001) with accuracy slightly higher in the orange-present condition, and a main effect of array size (*b* = −0.24, *SE* = 0.10, *z* = −2.44, *p* = 0.015) with a slight decrease of accuracy with increasing array size (Figure 3B).

Additionally, we analyzed sensitivity (d') and response bias (C) measures from Signal Detection Theory, in order to further characterize visual search performance in terms of hits and false alarms. Specifically, d' provides a measure of the ability to discriminate the target from the distractors (Verghese, 2001) while controlling for possible biases (C) in using one response more than the other (Palmer et al., 2000). For this analysis, standardized hit (Z_Hit_) and false alarm (Z_FA_) rates were computed as described above. The sensitivity index was defined as d' = ZHit – ZFA, while response bias was as above-defined C = −0.5^*^(Z_Hit_+Z_FA_). On each measure we separately conducted an ANOVA with target type and array size as within-subject factors and group as between-subject factor.

**Figure 4:**
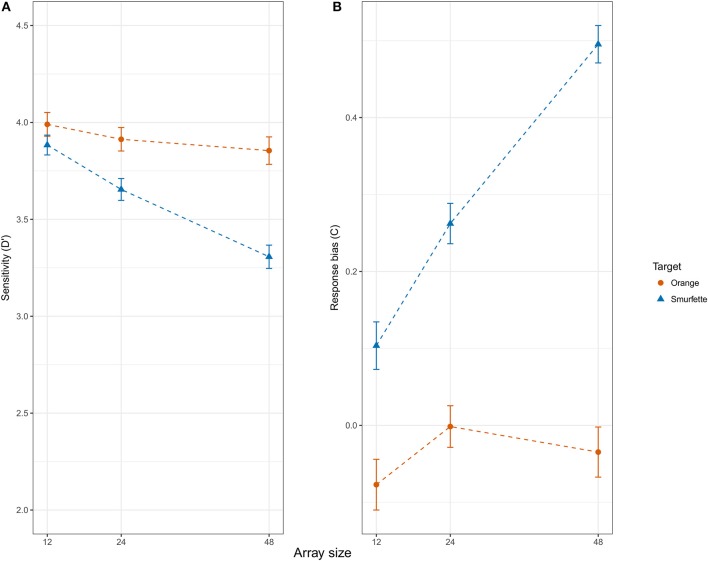
Signal detection theory (SDT) measures of sensitivity *d'* **(A)** and response bias *C* **(B)** as a function of target type (Orange vs. Smurfette) and array size (12, 24, 48). Error bars represent within-subjects standard errors of the mean (SEM; Morey, 2008).

The ANOVA on *d'* revealed significant effects of target type [*F*_(1, 45)_ = 28.48, *p* < 0.001, $\eta_{\text{p}}^{2}$ = 0.39], with a lower discriminability for Smurfette (*d*' = 3.61, *SE* = 0.06) as compared to orange (*d*' = 3.92, *SE* = 0.04), and array size [*F*_(2, 90)_ = 19.70, *p* < 0.001, $\eta_{\text{p}}^{2}$ = 0.30]. A Newman-Keuls' *post-hoc* test on the latter result revealed that discriminability was higher for the 12-item condition (*d*' = 3.92, *SE* = 0.05) as compared to the 24-item ones (d' = 3.90, *SE* = 0.06; *p* = 0.009) and for the latter condition as compared to the 48-item ones (*d*' = 3.75, *SE* = 0.06; *p* < 0.001). The analysis also revealed that this effect was modulated by target type [interaction: *F*_(2, 90)_ = 7.79, *p* < 0.001, $\eta_{\text{p}}^{2}$ = 0.15]. *Post-hoc* analyses showed that the above described effect of array size on discriminability was significant for Smurfette (12 > 24 > 48; *p*s < 0.015 and 0.001, respectively) but not for orange (12 = 24 = 48; *p*s > 0.339 and 0.748, respectively) (see Figure 4A). No other effect was significant.

The ANOVA on *C* yielded a similar pattern of results. Indeed, it revealed significant effects of target type [*F*_(1, 45)_ = 172.23, *p* < 0.001, $\eta_{\text{p}}^{2}$ = 0.79], with a conservative response bias for Smurfette (*C* = 0.29, *SE* = 0.02) as compared to orange (*C* = −0.04, *SE* = 0.02), and array size [*F*_(2, 90)_ = 25.72, *p* < 0.001, $\eta_{\text{p}}^{2}$ = 0.36]. *Post-hoc* analyses on the latter result revealed that the mean *C* value was lower for the 12-item condition (*C* = −0.06, *SE* = 0.02) as compared to the 24-item one (*C* = 0.05, *SE* = 0.03; *p* < 0.001) and for the latter condition as compared to the 48-item one (*C* = 0.11, *SE* = 0.02; *p* = 0.001). Again, the analysis revealed that this effect was modulated by target type [interaction: *F*_(2, 90)_ = 21.21, *p* < 0.001, $\eta_{\text{p}}^{2}$ = 0.32]. *Post-hoc* analyses showed that the above described effect of array size on *C* values was significant for Smurfette (12 < 24 < 48; both *p*s < 0.001) but not for orange (12 = 24 = 48; *p*s > 0.143 and 0.405, respectively) (see Figure 4B). No other effect was significant.

### Response times (RTS)

RTs were log-transformed to mitigate the influence of non-normal distribution and skewed data. Log-transformed RTs were analyzed by conducting a Linear Mixed Model (LMM) using the lmer function from the lme4 library (Bates et al., 2014). Error trials and post-error trials were excluded from the analysis. The full model (Equation 2) included all the fixed and random effects of the accuracy GLMM (with the exception of log-RTs). Moreover, to control for the RT temporal dependency between successive trials, we included as fixed effect the log-RT at the preceding trial (Baayen and Milin, 2010).

$$\begin{array}{l} logRT\;\sim trial\;+\;pre\text{\_}logRT\;+\;presence\;*\;target\;*\;array\;*\\ group+\left(1|id/c\right)+\left(0+trial|id\right) \end{array}$$

As for the accuracy model, *C* was introduced since individual's response bias can affect RTs, for example by causing faster responding to one condition than another. Initially, all the models were fitted using the Maximum Likelihood criterion to allow model comparisons (Bates et al., 2014). The full model resulted the best-fitting model [$\chi_{\left(8\right)}^{2}$ = 72.52, *p* < 0.001]. Visual inspection of the residuals showed that the model was a bit stressed. As suggested by Baayen and Milin (2010), trials with absolute standardized residuals higher than 2.5 standard deviations were considered outliers and removed (1.55% of the trials). After removing outlier trials, all the models were refitted and compared using a likelihood ratio test, and again the full model resulted the best-fitting model (Table 2). This time, visual inspection of residual plots of the full model did not show any evident violation of homoscedasticity and normality.

**Table 2:** Model fit analysis for log RTs data.

	**Fixed effects**	**Model *df***	**Chisq (*df*)**	***p***	**AIC**	**ΔAIC**	**RL**
M0	Intercept	5			16,169		
M1	Trial	6	25.08 (1)	<0.001	16,146	23.08	102,492
M2	Trial + pre_logRT	7	168.36 (1)	<0.001	15,980	166.36	>10^36^
M3	Trial + pre_logRT + presence	8	9949.45 (1)	<0.001	6,032	9947.45	>10^307^
M4	Trial + pre_logRT + presence ^*^ target	10	564.25 (2)	<0.001	5,472	560.25	>10^121^
M5	Trial + pre_logRT + presence ^*^ target ^*^ array	14	3587.81 (4)	<0.001	1,892	3579.81	>10^121^
M6	Trial + pre_logRT + presence ^*^ target ^*^ array ^*^ group	22	91.03 (8)	<0.001	1,817	75.03	>10^16^

*The table shows model comparison statistics. Fixed effects and degrees of freedom (df) are reported for each model. Models were fitted using maximum likelihood. Chi-squared and degrees of freedom, Chisq (df), and probability value, p, are based on likelihood ratio test between successive models. ΔAIC indicates the difference in Akaike Information Criterion between the model M(n−1) and the model M(n) [e.g., for the model M3, ΔAIC = AIC(M2) – AIC(M3)]. To compare the relative evidence of each model, we computed the relative likelihood, RL = exp(ΔAIC/2)*.

**Figure 5:**
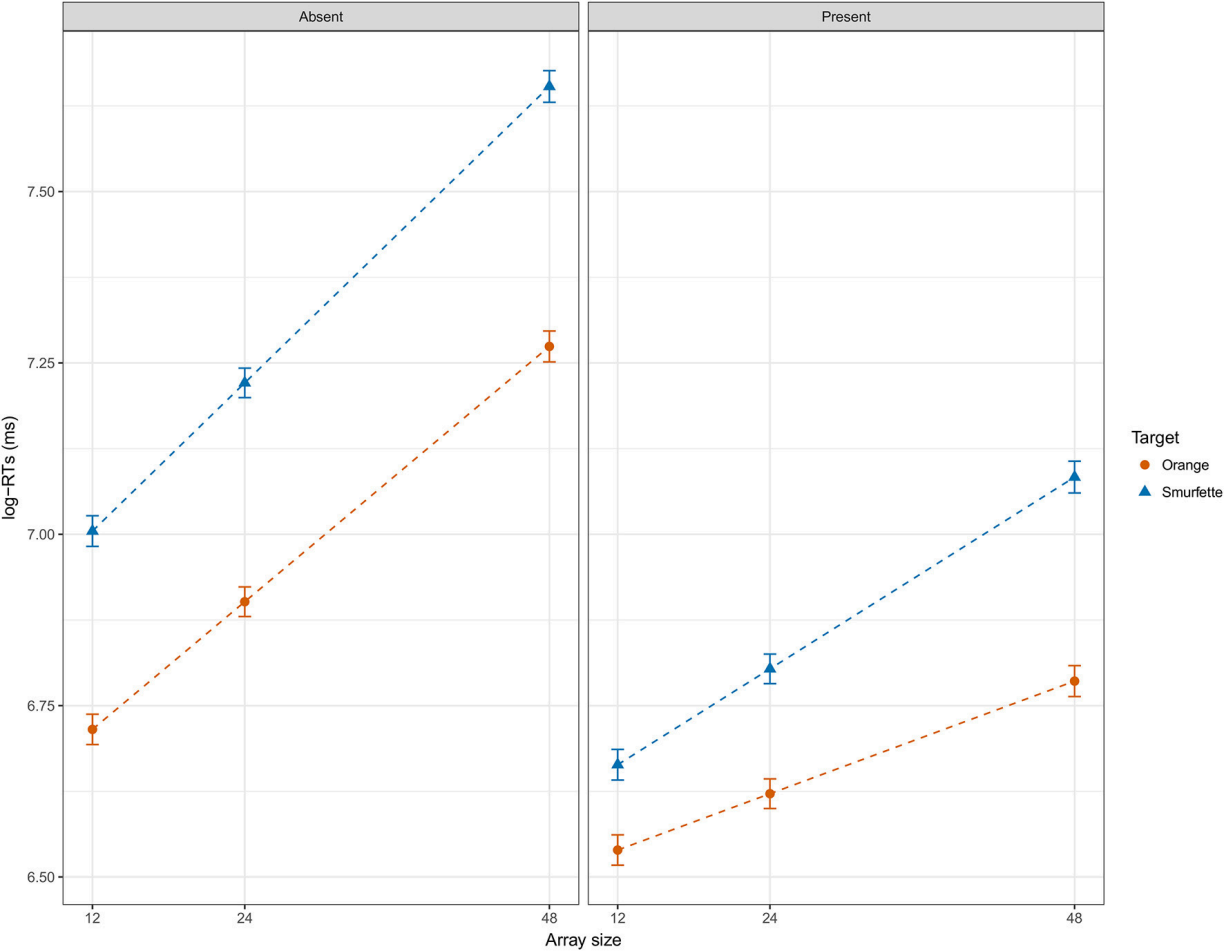
Effect display for the interaction of presence, target, and array size in the full LMM (REML) fit to log-RT data, and averaged over groups. Error bars represent standard errors of the estimated effect.

**Figure 6:**
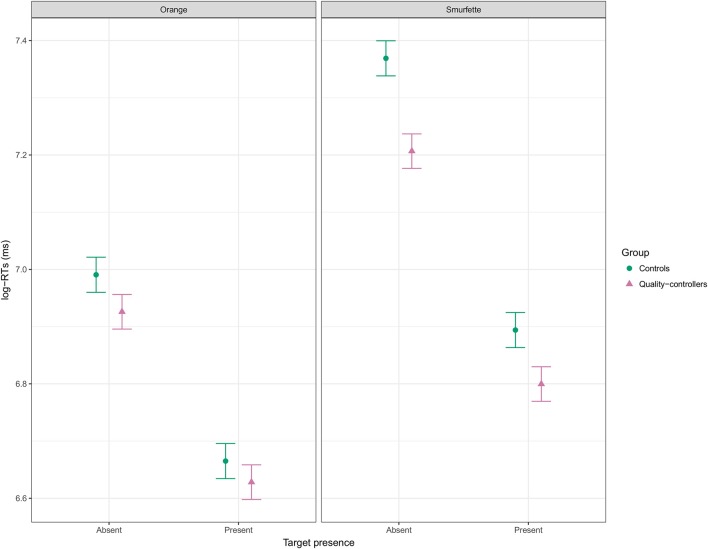
Effect display for the interaction of presence, target, and group in the full LMM (REML) fit to log-RT data, and averaged over array size. Error bars represent standard errors of the estimated effect.

At this point, the full model was refitted by minimizing the REML (Restricted Maximum Likelihood) criterion, as suggested by Bates (2014; see also Luke, 2017). Marginal *R*^2^ of the full model was 0.54 and conditional *R*^2^ was 0.69. Table 3 shows the statistical results of the type II ANOVA (as suggested by Langsrud, 2003) with additional F statistics based on Kenward-Roger's approximation of denominator degrees of freedom (Kenward and Roger, 1997). Overall, fitting mixed-effects models with REML and deriving *p*-values using Kenward-Roger's approximation seems to ensure optimal Type 1 error rate control (Luke, 2017). Figure 5 shows that RTs were longer in the Smurfette block compared to the orange one, and longer when both targets were absent. The effect of array size (i.e., an increase of RTs with increasing array size) was slightly lower for orange than for Smurfette, especially in the target-present condition (Figure 5). Concerning group differences (Figure 6), the increase in RTs in the Smurfette block (compared to the orange one) was much greater for controls than for quality-controllers. The effect of target presence (i.e., longer RTs in the target-absent condition compared to the target-present one) was greater for controls than for quality-controllers and this between-group difference was larger in the Smurfette block. To further investigate the three-way interaction between target presence, target type, and group variables two LMMs were fitted on the two task blocks separately. In the orange block, ANOVA results did not show any significant main effect of the group variable [*F*_(1, 64.5)_ = 1.8, *p* = 0.181, ß = −0.11], whereas there was a significant interaction between target presence and group [*F*_(1, 12620)_ = 11.8, *p* < 0.001, ß = 0.03]. Indeed, as shown in the Figure 6, quality-controllers were faster than controls only in the orange-absent condition. In the Smurfette block, there was a significant main effect of the group [*F*_(1, 74.1)_ = 6.3, *p* = 0.015, ß = −0.16], as well as a significant interaction effect between target presence and group [*F*_(1, 11952)_ = 52.3, *p* < 0.001, ß = 0.06]. In the Smurfette block, quality-controllers were faster than controls and this difference in RTs was more pronounced in the target-absent condition (Figure 6). No significant group difference was found to involve array size, with the exception of a three-way interaction between target type, array size, and group that was barely significant. To further explore this interaction, two LMMs were fitted for the absent/present condition separately. In both conditions no significant interaction between array size and group was found [target present: *F*_(1, 360)_ = 0.22, *p* = 0.638, ß = 0.02; target absent: *F*_(1, 1358.8)_ = 2.61, *p* = 0.106, ß = −0.02].

**Table 3:** Analysis of variance of log RTs data.

**Fixed effects**	**Sum Sq**	**Num. *df***	**Den. *df***	***F*-value**	***p***	**ß**
Time	2.85	1	96.0	46.9	<0.001	−0.133
Pre_logRT	14.85	1	24772.7	244.3	<0.001	−0.065
Presence	873.80	1	24610.7	14372.8	<0.001	−0.380
Target	38.85	1	665.9	639.0	<0.001	0.439
Array	184.60	1	1150.8	3036.4	<0.001	0.545
Group	0.28	1	49.5	4.6	=0.037	−0.075
Presence × target	25.45	1	24606.4	418.7	<0.001	−0.146
Presence × array	78.27	1	24606.8	1287.4	<0.001	−0.219
Target × array	3.53	1	1109.8	58.0	<0.001	0.068
Presence × group	3.49	1	24610.8	57.3	<0.001	0.028
Target × group	0.86	1	674.6	14.1	<0.001	−0.098
Array × group	0.05	1	1188.5	0.8	= 0.372	−0.012
Presence × target × array	1.83	1	24611.5	30.1	<0.001	0.039
Presence × target × group	0.62	1	24606.7	10.2	= 0.001	0.030
Presence × array × group	0.25	1	24606.7	4.0	= 0.044	0.015
Target × array × group	0.03	1	1138.8	0.6	= 0.455	−0.012
Presence × target × array × group	< 0.01	1	24612.1	<0.1	= 0.957	−0.001

*The table shows type III sums of squares. F-statistics and associated p-values were calculated using Kenward-Roger's approximation of degrees of freedom. Additionally, standardized regression coefficients (ß) are shown*.

### Image analysis

Differences in RTs and search slopes between orange-present and Smurfette-present conditions were not expected a priori. Since these findings could be likely explained by low-level visual properties, we compared the distinctiveness of the two targets among their distractors. The Adaptive Whitening Salience (AWS) model (Garcia-Diaz et al., 2012) was used to estimate the perceptual salience of each target. AWS is a bottom-up saliency model that provides maps of the predicted probability for each location in an image of being fixated on the basis of its low-level visual features. Notably, this model has shown to outperform important saliency models in predicting human eye fixations and in reproducing several psychophysical phenomena (Borji et al., 2013). For each target, we generated 144 images representing search displays, one for each combination of array size by target position. Saliency maps of these images were then computed using the authors' Matlab implementation of the AWS model. From each map, a target salience score was obtained by averaging the values of the points corresponding to the target location. Target scores were finally compared using a paired-sample *t*-test. Results did not show any significant difference between the two targets [*t*_(143)_ = 0.05, *p* = 0.963]. Since the saliency analysis did not explain the orange-present advantage, we compared perceptual similarity between the two targets and their distractors. Indeed, previous findings have shown that visual search difficulty increases with increased target-distractor perceptual similarity (Duncan and Humphreys, 1989). Usually, distinguishing real-world objects from one another is not easy on the basis of low-level visual features. However, it is not the case of the orange, which is highly characterized by its color. As with AWS, saliency maps for the 144 search displays were computed on the basis of a frequency-tuned approach (Achanta et al., 2009) that estimates saliency maps using color and luminance features. This method has been shown to outperform several state-of-the-art saliency models in object segmentation. Following the author's algorithm, images were Gaussian filtered and converted in the Lab color space. The L^*^a^*^b^*^ space is characterized by the luminance channel (L), a green-red opponent channel (a), and a blue-yellow opponent channel (b). This color space is preferable for its biological plausibility (Engel et al., 1997). Since selection of our stimuli was performed controlling for luminance, for each image the saliency map was computed finding the Euclidean distance between the a^*^b^*^ pixel vector and the average a^*^b^*^ vector. Saliency maps were min-max-scaled and target saliency scores were computed and compared as for the AWS model. Results showed that orange saliency was significantly higher than Smurfette saliency [*t*_(143)_ = 112.06, *p* < 0.001]. Similar results were obtained by computing saliency maps as the Euclidean distance between the L^*^a^*^b^*^ pixel vector and the average L^*^a^*^b^*^ vector [*t*_(143)_ = 57.34, *p* < 0.001].

## Discussion

The goal of the present study was to investigate whether intense professional visual search experience could enhance monitoring processes involved in visual search. To achieve this aim, we compared performance of a group of professional searchers (i.e., quality-controllers) with that of a well-matched control group on a computerized visual search task. The a priori hypothesis was to find an advantage for quality-controllers in those situations in which monitoring is especially involved, that is, when looking for the presence/absence of the target requires a more extensive evaluation of the search array (Vallesi, 2014).

To facilitate the discussion of the main results, it seems worthwhile to see how the overall performance pattern on the task was. All participants were slower in the target-absent condition, that is, the condition that was expected to rely much more on monitoring processes. Moreover, checking for the presence/absence of the target was more difficult in the Smurfette block, as revealed by longer RTs for both Smurfette-present/absent conditions (compared to the orange ones), and by the lower Smurfette discriminability. This difference in search efficiency for the two targets was not predicted a priori. Looking at the two target-present conditions, slopes of RTs as a function of array size (a measure often associated with perceptual search efficiency, see Rauschenberger and Yantis, 2006) suggested that differences in performance between searching for the two targets could be accounted for by low-level visual features. In order to verify whether low-level visual properties could explain target differences in search efficiency, we analyzed the perceptual salience of each target among their distractors. Results showed that the color was a salient low-level visual feature that more easily distinguished the orange (compared to Smurfette) from distractors. Therefore, searching for an orange led to more efficient search, likely because its color was a distinctive feature (Wolfe, 1998; Liesefeld et al., 2016). Overall, these results suggest that bottom-up selection processes likely favored orange detection due to its perceptual properties, thus reducing the need of evaluating each item of the array. Conversely, searching for the Smurfette target led to a less efficient search accompanied by more extensive monitoring needed to exhaustively evaluate the search array.

Concerning between-group differences, the results were consistent with our a priori hypothesis. Indeed, quality-controllers were faster than controls in the target-absent condition, the condition that, as discussed above, relied much more on monitoring. Moreover, this quality-controllers' advantage in the target-absent condition was more pronounced in the Smurfette block. This result is consistent with the fact that determining the absence of the Smurfette target required a more exhaustive evaluation (i.e., monitoring) of the search array compared to the orange target, as reflected by the general difference in RTs between the two target-absent conditions.

The target-present condition revealed two unexpected findings. First, according to studies on visual expertise (Hershler and Hochstein, 2009; Golan et al., 2014; Reeder et al., 2016), we expected to find a quality-controllers' advantage in detecting an object of expertise. However, no significant between-group difference emerged in the orange-present condition. One possible explanation for this negative finding could be a floor effect. However, the increase in RTs as a function of the array size makes this explanation unlikely. Indeed, even if it is plausible to explain the lack of a significant between-group difference as due to a floor effect in the 12-object array size, where RTs were indeed at their minimum, there was room for observing a quality-controllers' advantage in the higher array sizes, since RTs were longer in those conditions. A second explanation for this unexpected result could be that searching for the orange in our task mainly involved low-level visual mechanisms. Indeed, a similar result was found in a previous study with car experts performing a visual search task similar to ours (Golan et al., 2014). In that study, the authors found higher search efficiency for airplane targets than cars and butterflies across all groups involved. Remarkably, car experts exhibited no difference in search efficiency between their objects of expertise (i.e., cars) and airplanes. Even in that case, the authors explained efficient search for airplane in terms of discriminative perceptual features used by low-level visual mechanisms and largely independent of expertise with the target. A third non-mutually exclusive explanation could be that, since the orange is a more familiar target than Smurfette, it is possible that orange familiarity led to a more efficient search in both groups (Mruczek and Sheinberg, 2005; but see: Wang et al., 1994; Shen and Reingold, 2001).

The other unexpected result was the quality-controllers' advantage in detecting the Smurfette target, an object not related to their expertise. However, since searching for the Smurfette-target required monitoring to a greater extent, this quality-controllers' advantage was congruent with our hypothesis of a professional search-experience boost of top-down control processes, even in the absence of objects of expertise. Overall, between-group differences emerged in those situations that required a more extensive employment of monitoring processes.

An alternative interpretation of between-group differences in search efficiency could be in terms of quality-controllers' enhancement in general response speed (Castel et al., 2005). However, the lack of differences in the orange-present condition makes this interpretation implausible. Indeed, the experts' advantages emerged only in the hard situations (i.e., lower search efficiency), that is when their trained ability (i.e., monitoring) was likely required. In this regard, our results are consistent with recent studies showing that cognitive control can be shaped by immersive real-life training (e.g., Yildiz et al., 2014; Babcock and Vallesi, 2015; Arbula et al., 2016).

In summary, quality-controllers were faster in those conditions that extensively required monitoring processes. Moreover, differences between quality-controllers and controls were independent of visual expertise with the targets (e.g., expertise for oranges). These findings extend previous research on visual search and expertise, highlighting the importance of control processes in search performance. The present results provide evidence that top-down processes in visual search can be enhanced through extensive professional search experience beyond visual expertise specific advantages.

## Author contributions

AVi drafted the manuscript. He implemented the task, was involved in data collection and performed statistical analysis. AVa was involved in the conception of the work and provided ongoing contributions and feedback throughout the experimental process. He also provided additional revisions to the manuscript. All the authors have approved the final version of the manuscript and agree to be accountable for all aspects of the work.

### Conflict of interest statement

The authors declare that the research was conducted in the absence of any commercial or financial relationships that could be construed as a potential conflict of interest.
